# 2-{[(Pyrazin-2-yl)amino]­meth­yl}phenol

**DOI:** 10.1107/S1600536812031339

**Published:** 2012-07-18

**Authors:** Shan Gao, Seik Weng Ng

**Affiliations:** a Key Laboratory of Functional Inorganic Material Chemistry, Ministry of Education, Heilongjiang University, Harbin 150080, People’s Republic of China; b Department of Chemistry, University of Malaya, 50603 Kuala Lumpur, Malaysia; c Chemistry Department, Faculty of Science, King Abdulaziz University, PO Box 80203 Jeddah, Saudi Arabia

## Abstract

The two aromatic rings of the title compound, C_11_H_11_N_3_O, are nearly perpendicular to one another, with a dihedral angle between their planes of 80.52 (18)°. In the crystal, the amino N atom is a hydrogen-bond donor to the pyrazine N^1^ atom of an inversion-related mol­ecule and the hy­droxy O atom is a hydrogen-bond donor to the pyrazine N^4^ atom of another mol­ecule. The two hydrogen bonds lead to the formation of a helical chain that runs along the *b* axis.

## Related literature

For the related compound 2-(anilinometh­yl)phenol, see: Qu *et al.* (2007 ▶).
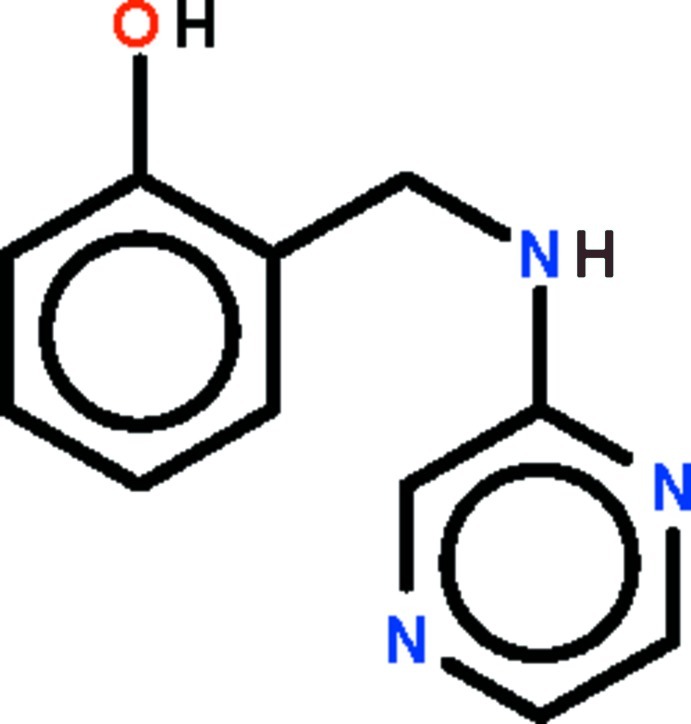


## Experimental

### 

#### Crystal data

C_11_H_11_N_3_O
*M* *_r_* = 201.23
Monoclinic, 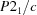

*a* = 9.7021 (14) Å
*b* = 13.0937 (17) Å
*c* = 7.8806 (13) Å
β = 95.746 (5)°
*V* = 996.1 (3) Å^3^
*Z* = 4
Mo *K*α radiation
μ = 0.09 mm^−1^
*T* = 295 K
0.26 × 0.22 × 0.17 mm

#### Data collection

Rigaku R-AXIS RAPID IP diffractometer
Absorption correction: multi-scan (*ABSCOR*; Higashi, 1995 ▶) *T*_min_ = 0.977, *T*_max_ = 0.985
7686 measured reflections
1751 independent reflections
858 reflections with *I* > 2σ(*I*)
*R*_int_ = 0.079

#### Refinement

*R*[*F* ^2^ > 2σ(*F* ^2^)] = 0.061
*wR*(*F* ^2^) = 0.191
*S* = 1.06
1751 reflections
144 parameters
2 restraints
H atoms treated by a mixture of independent and constrained refinement
Δρ_max_ = 0.18 e Å^−3^
Δρ_min_ = −0.20 e Å^−3^

### 

Data collection: *RAPID-AUTO* (Rigaku, 1998 ▶); cell refinement: *RAPID-AUTO*; data reduction: *CrystalClear* (Rigaku/MSC, 2002 ▶); program(s) used to solve structure: *SHELXS97* (Sheldrick, 2008 ▶); program(s) used to refine structure: *SHELXL97* (Sheldrick, 2008 ▶); molecular graphics: *X-SEED* (Barbour, 2001 ▶); software used to prepare material for publication: *publCIF* (Westrip, 2010 ▶).

## Supplementary Material

Crystal structure: contains datablock(s) global, I. DOI: 10.1107/S1600536812031339/xu5580sup1.cif

Structure factors: contains datablock(s) I. DOI: 10.1107/S1600536812031339/xu5580Isup2.hkl

Supplementary material file. DOI: 10.1107/S1600536812031339/xu5580Isup3.cml

Additional supplementary materials:  crystallographic information; 3D view; checkCIF report

## Figures and Tables

**Table 1:** Hydrogen-bond geometry (Å, °)

*D*—H⋯*A*	*D*—H	H⋯*A*	*D*⋯*A*	*D*—H⋯*A*
O1—H1⋯N2^i^	0.84 (1)	1.96 (1)	2.796 (4)	174 (4)
N3—H3⋯N1^ii^	0.89 (1)	2.12 (1)	3.007 (4)	175 (3)

Symmetry codes: (i) 
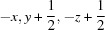
; (ii) 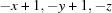.

**Fig. 1.**
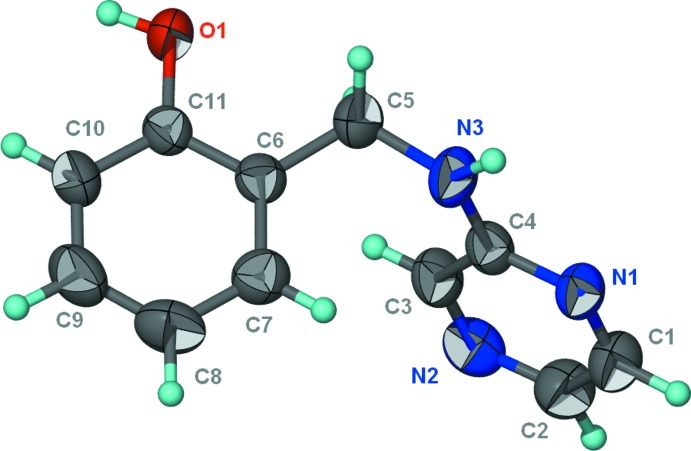
Thermal ellipsoid plot (Barbour, 2001) of C11H11N3O at the 50% probability level; hydrogen atoms are drawn as spheres of arbitrary radius.

**Fig. 2.**
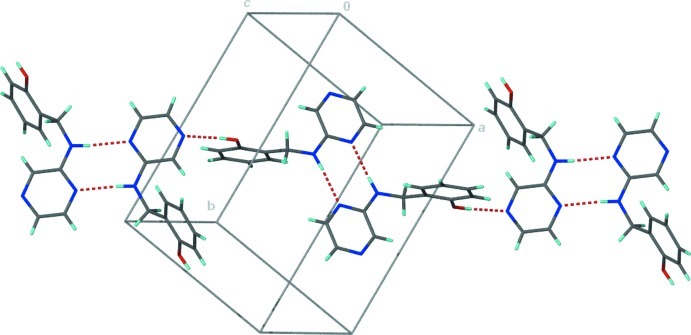
Hydrogen-bonded chain motif.

## supplementary crystallographic information

### Comment

Salicylaldehyde condenses with aromatic amines to yield Schiff bases, which serve as chelating ligands to a plethora of metal systems. These Schiff bases can be readily reduce to the corresponding secondary amines, which can also function as chelating ligands. Curiously, there are only few 2-(arylamino)methylphenols compared with the plethora of Schiff bases in the chemical literature. In 2-(anilinomethyl)phenol, the parent homolog, the hydroxy O atom is hydrogen-bonded to the amino N atom; another N–H···O hydrogen bond generates a dimer (Qu *et al.*, 2007). The two aromatic rings of the reduced Schiff-base, C_11_H_11_N_3_O (Scheme I), are twisted along the –CH_2_–NH– single-bond by 80.5 (1) °. The presence of basic sites allows for additional hydrogen-bonding interactions. The amino N atom is hydrogen-bond donor to the pyraziny-N^2^ atom of an inversion-related molecule and the hydroxy O atom is hydrogen-bond donor to the pyrazinyl-N^4^ atom another molecule. The two hydrogen bonds lead to the formation of a helical chain that runs along the *b*-axis of the monoclinic unit cell (Fig. 1, Table 1).

### Experimental

A solution of 2-aminopyrazine (1 mmol) and salicylaldehyde (1 mmol) in toluene (50 ml) was heated for 10 h. The solvent was removed under vacuum, and the residue was reduced in absolute methanol by sodium borohydride. Colorless crystals were obtained by recrystallization from methanol in 75% yield.

### Refinement

Carbon-bound H-atoms were placed in calculated positions (C–H 0.93 Å) and were included in the refinement in the riding model approximation, with *U*(H) set to 1.2*U*(C). The amino and hydroxy H-atoms were located in a difference Fourier map, and were refined with distance restraints N–H 0.88±0.01 Å, O–H 0.84±0.01 Å; their temperature factors were refined.

Although the crystal was measured up to a 2θ limit of 55 °, only the reflections below 50 ° were used for refinement owing to weak diffraction.

### Crystal data

**Table:**

C_11_H_11_N_3_O	*F*(000) = 424
*M**_r_* = 201.23	*D*_x_ = 1.342 Mg m^−^^3^
Monoclinic, *P*2_1_/*c*	Mo *K*α radiation, λ = 0.71073 Å
Hall symbol: -P 2ybc	Cell parameters from 3450 reflections
*a* = 9.7021 (14) Å	θ = 3.0–27.5°
*b* = 13.0937 (17) Å	µ = 0.09 mm^−^^1^
*c* = 7.8806 (13) Å	*T* = 295 K
β = 95.746 (5)°	Prism, colorless
*V* = 996.1 (3) Å^3^	0.26 × 0.22 × 0.17 mm
*Z* = 4	

### Data collection

**Table:**

Rigaku R-AXIS RAPID IP diffractometer	1751 independent reflections
Radiation source: fine-focus sealed tube	858 reflections with *I* > 2σ(*I*)
Graphite monochromator	*R*_int_ = 0.079
ω scan	θ_max_ = 25.0°, θ_min_ = 3.0°
Absorption correction: multi-scan (*ABSCOR*; Higashi, 1995)	*h* = −11→11
*T*_min_ = 0.977, *T*_max_ = 0.985	*k* = −15→15
7686 measured reflections	*l* = −9→9

### Refinement

**Table:**

Refinement on *F*^2^	Primary atom site location: structure-invariant direct methods
Least-squares matrix: full	Secondary atom site location: difference Fourier map
*R*[*F*^2^ > 2σ(*F*^2^)] = 0.061	Hydrogen site location: inferred from neighbouring sites
*wR*(*F*^2^) = 0.191	H atoms treated by a mixture of independent and constrained refinement
*S* = 1.06	*w* = 1/[σ^2^(*F*_o_^2^) + (0.0862*P*)^2^ + 0.1018*P*] where *P* = (*F*_o_^2^ + 2*F*_c_^2^)/3
1751 reflections	(Δ/σ)_max_ = 0.001
144 parameters	Δρ_max_ = 0.18 e Å^−^^3^
2 restraints	Δρ_min_ = −0.20 e Å^−^^3^

### Fractional atomic coordinates and isotropic or equivalent isotropic displacement parameters (Å^2^)

**Table:**

	*x*	*y*	*z*	*U*_iso_*/*U*_eq_	
O1	−0.0468 (3)	0.63270 (19)	0.2017 (3)	0.0656 (8)	
N1	0.4697 (3)	0.3661 (2)	0.0705 (4)	0.0578 (8)	
N2	0.2758 (3)	0.2358 (2)	0.1926 (4)	0.0691 (9)	
N3	0.3227 (3)	0.5014 (2)	0.0842 (4)	0.0654 (9)	
C1	0.4925 (4)	0.2662 (3)	0.0949 (5)	0.0684 (11)	
H1A	0.5772	0.2396	0.0707	0.082*	
C2	0.3983 (4)	0.2013 (3)	0.1530 (5)	0.0724 (12)	
H2	0.4193	0.1322	0.1654	0.087*	
C3	0.2500 (4)	0.3333 (3)	0.1698 (5)	0.0650 (10)	
H3A	0.1651	0.3589	0.1953	0.078*	
C4	0.3470 (3)	0.4010 (3)	0.1077 (4)	0.0530 (9)	
C5	0.1935 (4)	0.5509 (3)	0.1131 (5)	0.0642 (10)	
H5A	0.1176	0.5081	0.0658	0.077*	
H5B	0.1875	0.6149	0.0508	0.077*	
C6	0.1742 (3)	0.5727 (2)	0.2970 (5)	0.0521 (9)	
C7	0.2751 (4)	0.5529 (3)	0.4289 (5)	0.0649 (11)	
H7	0.3577	0.5226	0.4051	0.078*	
C8	0.2562 (5)	0.5771 (3)	0.5957 (6)	0.0791 (13)	
H8	0.3241	0.5620	0.6839	0.095*	
C9	0.1347 (5)	0.6239 (3)	0.6285 (5)	0.0824 (13)	
H9	0.1218	0.6427	0.7396	0.099*	
C10	0.0329 (4)	0.6431 (3)	0.5000 (5)	0.0697 (11)	
H10	−0.0494	0.6737	0.5242	0.084*	
C11	0.0521 (4)	0.6171 (2)	0.3347 (5)	0.0524 (9)	
H1	−0.116 (2)	0.661 (2)	0.239 (5)	0.077 (13)*	
H3	0.388 (3)	0.538 (2)	0.040 (4)	0.070 (12)*	

### Atomic displacement parameters (Å^2^)

**Table:**

	*U*^11^	*U*^22^	*U*^33^	*U*^12^	*U*^13^	*U*^23^
O1	0.0572 (17)	0.0852 (18)	0.0555 (17)	0.0224 (13)	0.0117 (14)	−0.0003 (13)
N1	0.0494 (18)	0.0610 (19)	0.065 (2)	0.0002 (13)	0.0146 (15)	0.0000 (15)
N2	0.071 (2)	0.071 (2)	0.065 (2)	−0.0179 (17)	0.0064 (19)	0.0054 (16)
N3	0.054 (2)	0.066 (2)	0.080 (2)	0.0043 (16)	0.0281 (18)	0.0083 (17)
C1	0.060 (2)	0.069 (3)	0.078 (3)	0.0030 (19)	0.015 (2)	−0.002 (2)
C2	0.074 (3)	0.066 (2)	0.078 (3)	−0.004 (2)	0.010 (2)	0.004 (2)
C3	0.051 (2)	0.082 (3)	0.064 (3)	−0.0058 (19)	0.017 (2)	0.004 (2)
C4	0.046 (2)	0.066 (2)	0.048 (2)	−0.0072 (16)	0.0078 (17)	−0.0019 (17)
C5	0.054 (2)	0.077 (2)	0.064 (3)	0.0078 (18)	0.016 (2)	0.0054 (19)
C6	0.051 (2)	0.0549 (19)	0.051 (2)	−0.0027 (16)	0.0072 (18)	0.0043 (16)
C7	0.055 (2)	0.071 (2)	0.068 (3)	−0.0020 (18)	0.001 (2)	0.001 (2)
C8	0.087 (3)	0.084 (3)	0.063 (3)	−0.006 (2)	−0.015 (3)	0.004 (2)
C9	0.112 (4)	0.087 (3)	0.049 (3)	0.007 (3)	0.008 (3)	−0.012 (2)
C10	0.084 (3)	0.077 (3)	0.050 (3)	0.016 (2)	0.017 (2)	−0.001 (2)
C11	0.058 (2)	0.051 (2)	0.049 (2)	0.0017 (16)	0.0087 (19)	0.0025 (16)

### Geometric parameters (Å, º)

**Table:**

O1—C11	1.363 (4)	C5—C6	1.507 (5)
O1—H1	0.844 (10)	C5—H5A	0.9700
N1—C4	1.336 (4)	C5—H5B	0.9700
N1—C1	1.337 (4)	C6—C7	1.379 (5)
N2—C3	1.311 (4)	C6—C11	1.379 (5)
N2—C2	1.337 (4)	C7—C8	1.382 (5)
N3—C4	1.345 (4)	C7—H7	0.9300
N3—C5	1.449 (4)	C8—C9	1.375 (6)
N3—H3	0.888 (10)	C8—H8	0.9300
C1—C2	1.361 (5)	C9—C10	1.365 (5)
C1—H1A	0.9300	C9—H9	0.9300
C2—H2	0.9300	C10—C11	1.377 (5)
C3—C4	1.415 (5)	C10—H10	0.9300
C3—H3A	0.9300		
			
C11—O1—H1	109 (3)	N3—C5—H5B	108.4
C4—N1—C1	116.2 (3)	C6—C5—H5B	108.4
C3—N2—C2	117.3 (3)	H5A—C5—H5B	107.5
C4—N3—C5	123.9 (3)	C7—C6—C11	118.5 (3)
C4—N3—H3	117 (2)	C7—C6—C5	122.8 (3)
C5—N3—H3	119 (2)	C11—C6—C5	118.6 (3)
N1—C1—C2	123.6 (3)	C6—C7—C8	121.4 (4)
N1—C1—H1A	118.2	C6—C7—H7	119.3
C2—C1—H1A	118.2	C8—C7—H7	119.3
N2—C2—C1	120.7 (4)	C9—C8—C7	118.6 (4)
N2—C2—H2	119.7	C9—C8—H8	120.7
C1—C2—H2	119.7	C7—C8—H8	120.7
N2—C3—C4	122.3 (3)	C10—C9—C8	120.9 (4)
N2—C3—H3A	118.9	C10—C9—H9	119.6
C4—C3—H3A	118.9	C8—C9—H9	119.6
N1—C4—N3	116.9 (3)	C9—C10—C11	120.0 (4)
N1—C4—C3	120.0 (3)	C9—C10—H10	120.0
N3—C4—C3	123.2 (3)	C11—C10—H10	120.0
N3—C5—C6	115.4 (3)	O1—C11—C10	122.6 (3)
N3—C5—H5A	108.4	O1—C11—C6	116.8 (3)
C6—C5—H5A	108.4	C10—C11—C6	120.5 (4)
			
C4—N1—C1—C2	0.4 (6)	N3—C5—C6—C11	−178.2 (3)
C3—N2—C2—C1	1.3 (6)	C11—C6—C7—C8	−0.4 (5)
N1—C1—C2—N2	−1.2 (7)	C5—C6—C7—C8	177.7 (3)
C2—N2—C3—C4	−0.7 (6)	C6—C7—C8—C9	−1.4 (6)
C1—N1—C4—N3	179.9 (3)	C7—C8—C9—C10	2.2 (6)
C1—N1—C4—C3	0.2 (5)	C8—C9—C10—C11	−1.2 (6)
C5—N3—C4—N1	177.9 (3)	C9—C10—C11—O1	178.8 (3)
C5—N3—C4—C3	−2.5 (6)	C9—C10—C11—C6	−0.7 (6)
N2—C3—C4—N1	−0.1 (6)	C7—C6—C11—O1	−178.0 (3)
N2—C3—C4—N3	−179.7 (4)	C5—C6—C11—O1	3.8 (4)
C4—N3—C5—C6	78.5 (5)	C7—C6—C11—C10	1.4 (5)
N3—C5—C6—C7	3.7 (5)	C5—C6—C11—C10	−176.7 (3)

### Hydrogen-bond geometry (Å, º)

**Table:**

*D*—H···*A*	*D*—H	H···*A*	*D*···*A*	*D*—H···*A*
O1—H1···N2^i^	0.84 (1)	1.96 (1)	2.796 (4)	174 (4)
N3—H3···N1^ii^	0.89 (1)	2.12 (1)	3.007 (4)	175 (3)

Symmetry codes: (i) −*x*, *y*+1/2, −*z*+1/2; (ii) −*x*+1, −*y*+1, −*z*.
